# Correction of a severe facial asymmetry with computerized planning and with the use of a rapid prototyped surgical template: a case report/technique article

**DOI:** 10.1186/1746-160X-10-27

**Published:** 2014-07-11

**Authors:** Laszlo Seres, Endre Varga, Andras Kocsis, Zoltan Rasko, Balazs Bago, Endre Varga, Jozsef Piffko

**Affiliations:** 1 Department of Oral and Maxillofacial Surgery, University of Szeged, Kalvaria sgt. 57, 6725 Szeged, Hungary; 2 Orthodontic Clinic, Marostoi u. 29/A, 6726 Szeged, Hungary; 3 Smart Dental Solutions Ltd, Pulcz u. 46/B, 6724 Szeged, Hungary; 4 Department of Traumatology, University of Szeged, Semmelweis u. 6, 6725 Szeged, Hungary

**Keywords:** Virtual model surgery, Facial asymmetry, Computer-aided surgery, Digital intermediate wafer, Three-dimensional rapid prototyping

## Abstract

Management of significant facial asymmetry presents a challenge due to the geometric complexity of the bony and other facial structures. Manual model surgery is an essential part of treatment planning but it can be complicated, time-consuming and may contain potential errors. Computer-aided surgery has revolutionized the correction of maxillofacial deformities. The aim of this study was to report a case of facial asymmetry when computerised simulation surgery was performed instead of manual model surgery and a virtually planned wafer splint was fabricated. A 26-year-old male was presented with a severe right-sided hemimandibular elongation. Following presurgical orthodontics high-resolution computer tomography scan was performed. The stack images were reformatted into a three-dimensional structure. Virtual Le Fort-I osteotomy was performed and the symmetry of the maxilla was corrected with the help of a three-dimensional planning software. A virtual intermediate surgical wafer was designed and produced with three-dimensional rapid prototyping technology. The mandible was rotated into the correct position following virtual bilateral sagittal split osteotomy to visualize the movements of the osteotomised mandibular segments. The two-jaw procedure was performed according to the virtual plan. The facial symmetry was improved significantly and stable occlusion was achieved. This complex case shows the advantages of computer-aided surgical planning and three-dimensional rapid prototyping for the correction of facial asymmetries.

## Background

Correction of severe facial asymmetry is a challenging task due to the geometric complexity of the dentition, the bony structures and the soft tissues. Mandibular asymmetry is usually associated with a unilateral vertical maxillary excess and an occlusal cant, therefore, in most cases the deformity cannot be treated with single-jaw surgery [1].

Traditional cephalometric analysis is of limited value in interpreting the cause of the asymmetry, because complex three-dimensional (3D) structures are projected onto two-dimensional (2D) planes. Treatment planning of an asymmetric case requires three-dimensional consideration in the sagittal, coronal and horizontal planes. Traditionally, manual model surgery is an essential part of the preoperative workup that involves many time-consuming laboratory based steps. When two-jaw surgery is performed, following the transposition of the maxilla on the stone dental model in the articulator an interocclusal splint is fabricated that serves as an intermediate guide for repositioning the maxilla relative to the intact mandible. The second, or final wafer relates the mobilized mandible to the fixated maxilla.

It is of critical importance that model surgery is based on accurate translation of the theoretical transposition data. Any discrepancy between the plan and the model surgery will lead to an inaccurate interocclusal splint. A poorly designed and/or fabricated wafer can lead to a disastrous outcome even when the most skillful surgical technique is used.

If a symmetric or slightly asymmetric face is operated on, when the jaws are moved mainly in the anteroposterior and vertical direction, traditional, 2D analysis and planning are usually satisfactory. But even in these cases, small errors in each step of model surgery can compound and lead to an inaccurate result [2-4].

Major asymmetry involving both the upper and lower jaws often requires complicated two-jaw surgery. In these cases 3D planning is essential. The problem is twofold: first, how the most precise planning can be achieved and second, how the treatment plan can be transferred to the operating room. Accurate cephalometric analyses and 3D planning based on plain lateral and frontal cephalograms are hardly possible [5-7].

Improved imaging techniques and advances in software engineering have moved 3D computer models from the research and development area into routine clinical application [8-10]. Three-dimensional reconstruction images can be easily rotated and viewed from any angle. Accurate measurements can be performed on the maxillofacial complex and this helps not only to understand the etiology of facial asymmetry but to plan the osteotomies and movements of the segments.

Rapid prototyping is a remarkable, quickly evolving technology that has been revolutionizing the manufacturing process in several fields. With these technologies splints can be made that can guarantee the precise repositioning of the bony segments during surgery [11,12].

The aim of this study is twofold, first, to investigate whether virtual 3D model surgery is suitable for treatment planning of an asymmetric two-jaw surgery, and second, to examine if rapid prototyping may eliminate the need for manual model surgery and the conventional fabrication of the interocclusal splint in the dental laboratory. A case of a severe facial asymmetry is reported when computer aided surgical planning was performed and the intermediate wafer was designed virtually and was manufactured by a three-dimensional printer.

## Case presentation/technique description

A 26-year-old male complained of facial asymmetry and eating difficulties. Clinical evaluation revealed severe right-sided hemimandibular elongation with small compensatory transverse canting of the maxillary occlusal plane (Figure 1).Cross bite was observed on the left side. In the sagittal plane Class III malocclusion was noted on the right side while Class I molar and canine relationship was found on the left side. The mandibular front teeth, the left sided premolars and molars were lingually inclined. The maxillary midline was coincident with the facial midline. The mandibular dental midline and the mentum deviated to the left 11.8 and 15 mm, respectively (Figures 2, 3 and 4). There was mild crowding in both arches. Technetium isotope failed to show increased uptake in the condylar regions.

**Figure 1:**
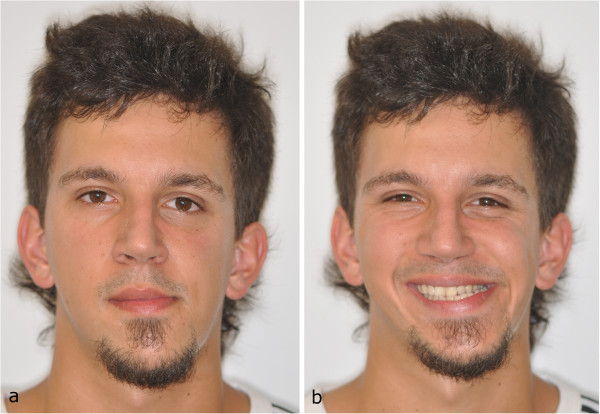
Initial facial view (a) and initial facial smiling (b).

**Figure 2:**
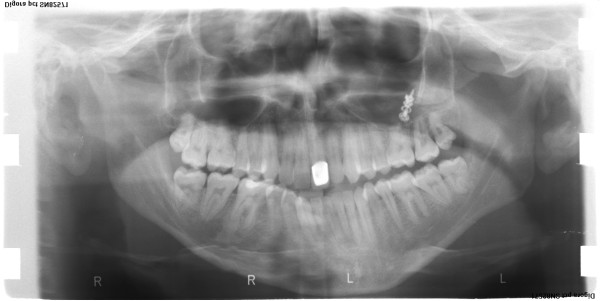
Initial panoramic radiograph.

**Figure 3:**
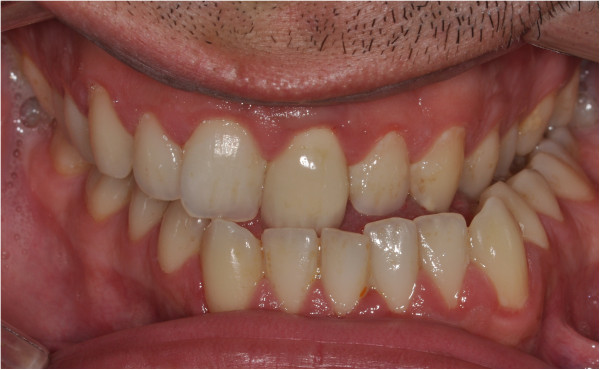
Pretreatment intraoral photograph.

**Figure 4:**
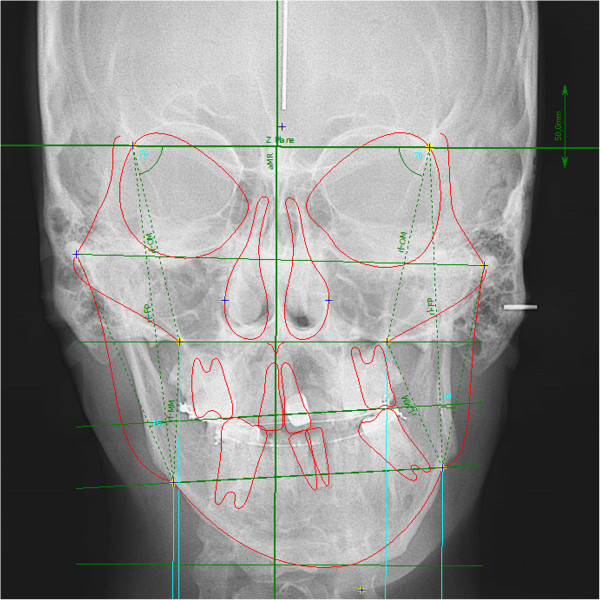
Presurgery frontal cephalometric x-ray shows the compensatory transverse canting of the maxillary occlusal plane.

Preoperative orthodontic treatment lasted for 18 months and consisted of alignment and elimination of the transverse and sagittal dental compensation and arch coordination.After presurgical orthodontic treatment was completed, computed tomography scan was obtained with an isotropic image resolution of 0.3 mm and standard image acquisition parameters (60kVp/40 keV (900 μA). The patient was scanned in a supine position. The gantry had a zero inclination. The digital imaging and communication in medicine (DICOM) data were directly transferred to a personal computer. An in-house developed 3D planning software (JMed software, TraumArt Ltd, University of Szeged, Hungary) was used to reformat DICOM stack images into a 3D structure and to perform virtual preoperative surgical planning (Figure 5a).Three-dimensional facial analysis was performed following the construction of the midsagittal, the Frankfort horizontal and the mandibular planes (Figure 5b). Maxillary height, ramus length, body length, body height, frontal ramal inclination and lateral ramal inclination were measured on both sides. After evaluation of the factors that contributed the chin deviation virtual surgery was performed.

**Figure 5:**
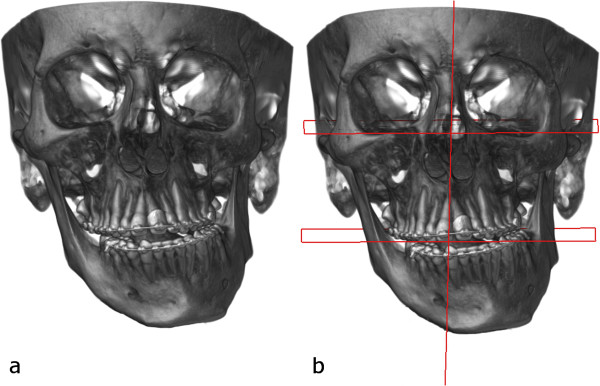
Preoperative CT scan (a) with planes defined (b).

Selection of the bony structures was made by the software’s semi-automatic segmentation tool and surface models were generated from each structure (Figure 6) [13,14]. Virtual conventional Le Fort I osteotomy was performed on the segmented models. The osteotomized segment was selected and was individually moved and rotated to reconstruct the symmetry of the maxilla (Figure 7a).A model of a virtual intermediate surgical wafer was created with the maxilla in the planned position and the mandible in its original place (Figure 7b). The model of the wafer was printed with a 3D printer from a bio-compatible synthetic material that is suitable for short-term mucosal-membrane contact (Figure 8). The splint was checked for occlusion on the patient’s lower and upper dental arches and fitted well in both cases.Following that the mandible was rotated into the correct position with virtual bilateral sagittal split osteotomy to visualise the movements of the osteotomized segments (Figure 9).In the surgical phase Le Fort I osteotomy was carried out as planned. The mobilized maxillary segment was rotated clockwise and was repositioned by application of the virtually planned intermediate wafer and mandibulomaxillary fixation. The virtually designated intermediate wafer fitted well during surgery (Figure 10). Miniplate fixation to the vertical facial buttresses was performed. Mandibulomaxillary fixation was then released and the bilateral sagittal split mandibular osteotomies were carried out. The distal segment of the mandible was rotated to the right and placed into the desired occlusion. Final splint was not used as the teeth were in good occlusion. Osteosynthesis was performed with titanium miniplates and screws (Figures 11 and 12). Postoperative intermaxillary fixation was maintained with tight elastics for 2 weeks. Loose guidance elastics were worn for a further period of 10 weeks. Two months after the surgical procedure, orthodontic treatment was resumed. Final arch coordination and occlusal settling were accomplished during the next 6 months (Figure 13). One year after the first surgery, genioplasty was performed at the same time of the removal of the previously implanted miniplates and screws. Standard intraoperative measurements were used without templates, the chin was rotated to the left by 4 mms (Figures 14 and 15).

**Figure 6:**
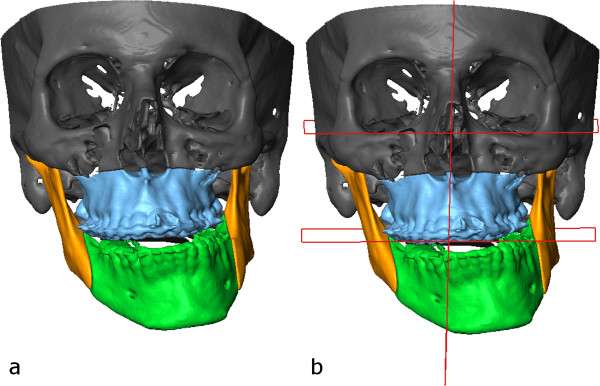
Selection of the bony structures (a) with planes defined (b).

**Figure 7:**
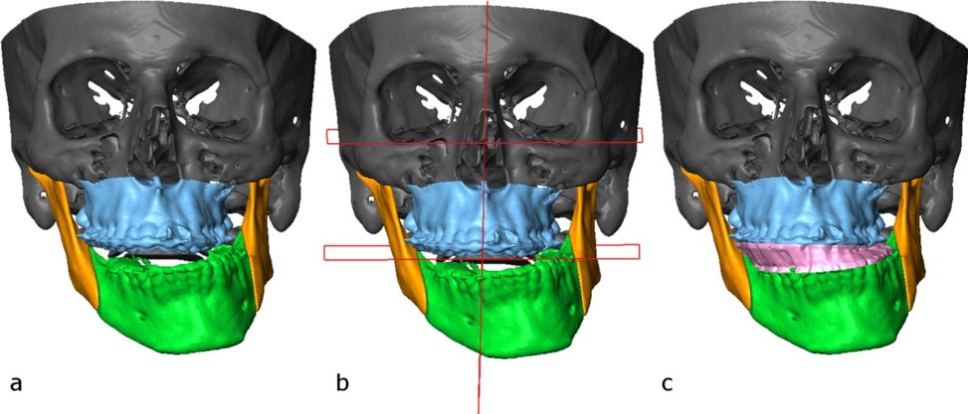
Virtually repositioned maxilla (a) with planes defined (b) and with the virtual intermediate wafer (c).

**Figure 8:**
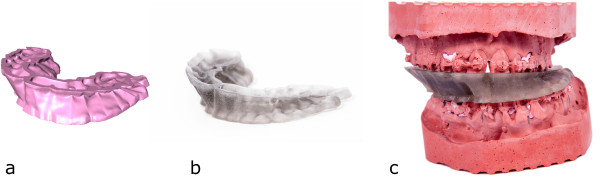
The virtual intermediate wafer (a) manufactured with 3D printer (b) and placed in the plaster cast (c).

**Figure 9:**
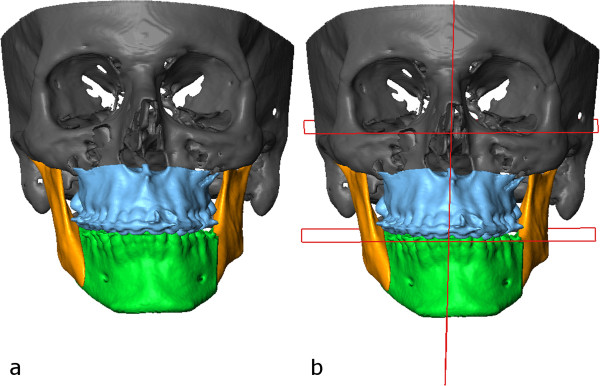
Virtually repositioned mandible (a) with planes defined (b).

**Figure 10:**
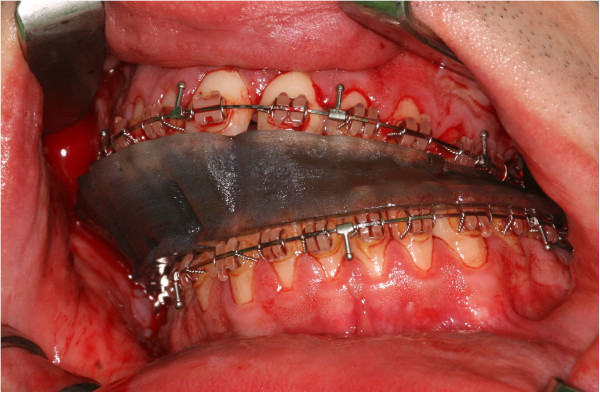
In surgery, the intermediate wafer fits well into the maxillary and mandibular dentition.

**Figure 11:**
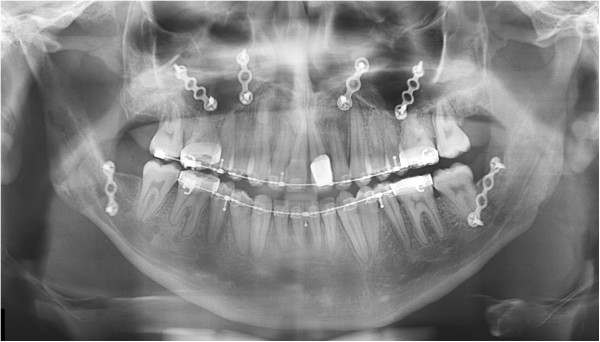
Panoramic radiograph after two-jaw surgery.

**Figure 12:**
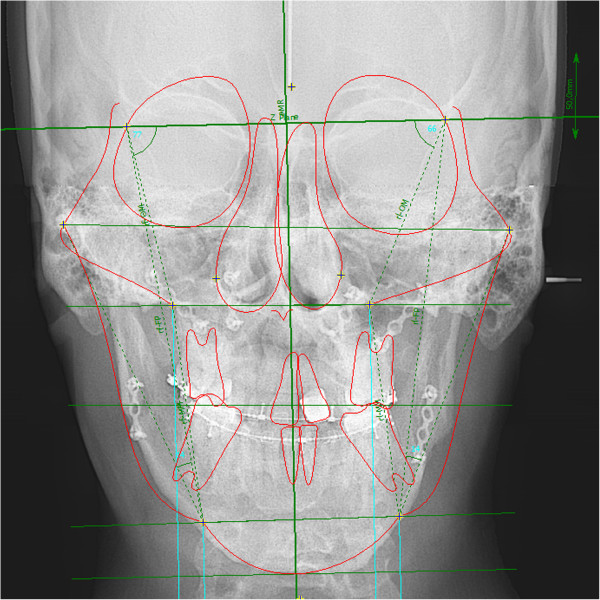
Postoperative frontal cephalometric x-ray shows that the facial symmetry has improved significantly.

**Figure 13:**
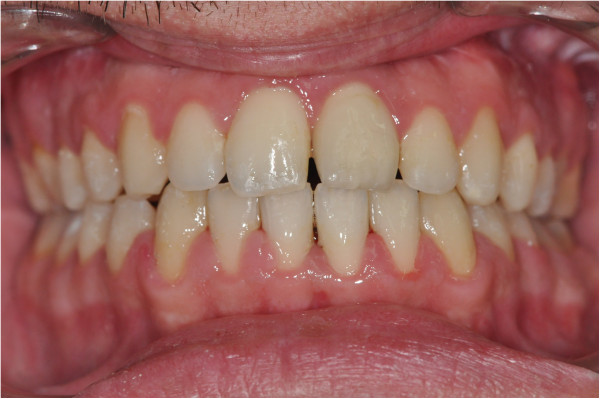
Final intraoral photograph.

**Figure 14:**
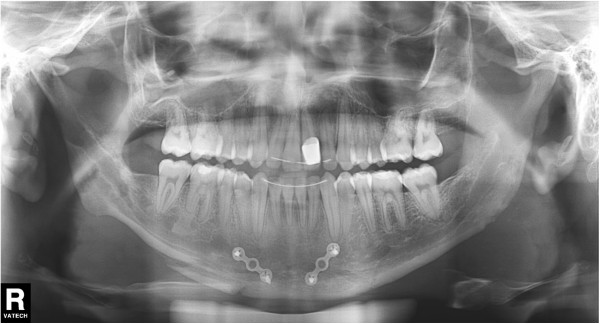
Final panoramic radiograph.

**Figure 15:**
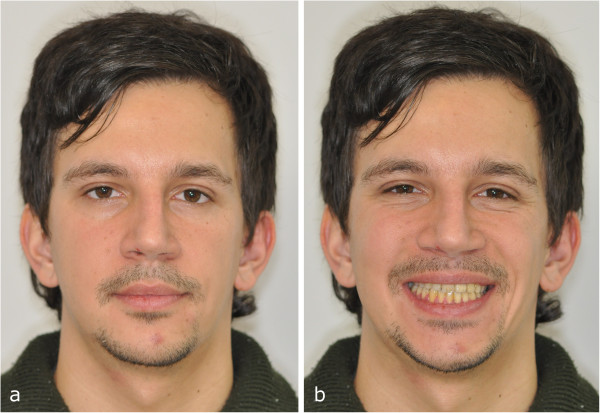
Final facial view (a) and final facial smiling (b).

The facial symmetry was improved significantly after the operations. The occlusion is stable and the patient is satisfied with his facial appearance. There is no sign of relapse after 18 months following the first surgical procedure (Figure 16).A postoperative CT scan was made to evaluate the difference between the virtual Le Fort I osteotomy and the surgical result. The preoperatively planned model of the segmented maxilla was superimposed on the postoperative CT scan. The distance map generated between the superimposed models shows only minimal deviations on the bony structures (Figure 17).

**Figure 16:**
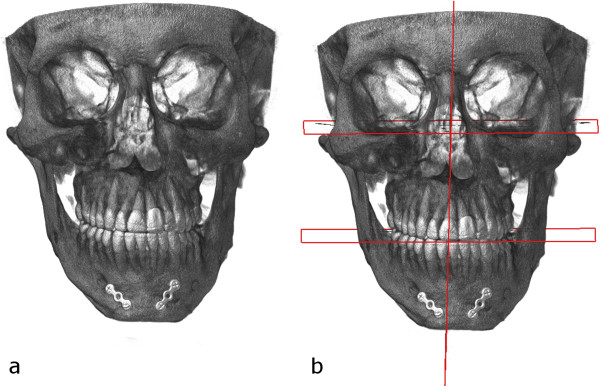
Postoperative CT scan (a) with planes defined (b).

**Figure 17:**
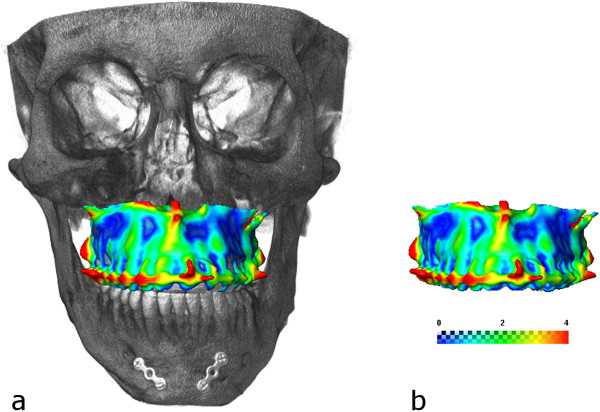
Superimposition of the virtual plan visualized on the skull (a) and with measurement (b).

## Discussion

When two-jaw surgery is planned for an asymmetric case spatial positioning of the maxilla is considered more critical than the repositioning of the mandible. Intermediate wafers are used more frequently than the final ones [15]. In our case reported, the key point of the procedure was the accurate repositioning of the maxilla. The maxillary segment mobilized via Le Fort I osteotomy showed acceptable intrinsic symmetry, therefore further maxillary osteotomies were not performed. The mobilized unit was placed symmetrically with respect to the sagittal plane. To achieve this it was rotated clockwise until the maxillary occlusal plane was parallel to the interpupillary line. At the same time a further rotation was performed in the horizontal plane to move the posterior part of the maxilla to the right. Amongst others, the complex rotational movement can be one of the sources of inaccuracies in manual model surgery. While with traditional methods treatment planning and model surgery are two separate steps, with virtual surgery these two procedures can be performed at the same time, there is no information loss between the two. The most complicated movements can be made precisely and the most accurate measurements can be calculated simultaneously. The final product of virtual model surgery is a virtual splint that can be materialized by rapid prototyping. This technique relies on the accuracy of the virtual model and the production of the surgical splint to ensure a successful surgery [16]. The intermediate wafer was fabricated with the highest printing accuracy in our case and it proved to be the most reliable tool to transfer virtual surgery into the operating room.

The new position of the mandible was determined by the repositioned maxilla. Virtual surgery showed that the mandible would be brought forward by 8.6 mms on the left side. Manual model surgery focuses on the dentition and the occlusion but changes in the bony structure cannot be displayed. Although final wafer was not used as the mandible was simply placed into maximal intercuspal occlusion, virtual surgery still helped us to understand and visualize the rotational movement of the mandible.

## Conclusions

Latest computerized and rapid prototyping technologies let us fully imagine, design and control orthognathic procedures without information loss among the surgeons, orthodontists and dental technicians. Any number of alternative treatment strategies can be investigated simultaneously during the planning phase. Computerised simulation surgery can be extremely useful in severe asymmetric cases when precise treatment planning with traditional methods is hardly possible. With this method manual model surgery and other laboratory steps can be avoided. The surgical wafer splint can be planned virtually and fabricated by a 3D printer.

## Consent

Written informed consent was obtained from the patient for publication of this Case report and any accompanying images. A copy of the written consent is available for review by the Editor-in-Chief of this journal.

## Competing interests

The authors declare that they have no competing interests.

## Authors' contributions

LS is responsible for the design and writing of the paper; he took part in surgical planning and performed surgery. EVJr and BB participated in writing and finalizing the paper, the software development and virtual surgical planning. AK performed orthodontics and was responsible for surgical planning. ZR took part in surgical planning and surgery. EV is the group leader of the software development team. JP is the group leader and helped in the revision of the manuscript. All authors read and approved the final manuscript.
